# Quantitative Histological Validation of Diffusion MRI Fiber Orientation Distributions in the Rat Brain

**DOI:** 10.1371/journal.pone.0008595

**Published:** 2010-01-07

**Authors:** Trygve B. Leergaard, Nathan S. White, Alex de Crespigny, Ingeborg Bolstad, Helen D'Arceuil, Jan G. Bjaalie, Anders M. Dale

**Affiliations:** 1 Centre for Molecular Biology and Neuroscience, Institute of Basic Medical Sciences, University of Oslo, Oslo, Norway; 2 Department of Cognitive Sciences, University of California San Diego, San Diego, California, United States of America; 3 A.A. Martinos Center for Biomedical Imaging, Massachusetts General Hospital, Harvard Medical School, Boston, Massachusetts, United States of America; 4 Departments of Neurosciences and Radiology, University of California San Diego, San Diego, California, United States of America; Cuban Neuroscience Center, Cuba

## Abstract

Diffusion MRI (dMRI) is widely used to measure microstructural features of brain white matter, but commonly used dMRI measures have limited capacity to resolve the orientation structure of complex fiber architectures. While several promising new approaches have been proposed, direct quantitative validation of these methods against relevant histological architectures remains missing. In this study, we quantitatively compare neuronal fiber orientation distributions (FODs) derived from *ex vivo* dMRI data against histological measurements of rat brain myeloarchitecture using manual recordings of individual myelin stained fiber orientations. We show that accurate FOD estimates can be obtained from dMRI data, even in regions with complex architectures of crossing fibers with an intrinsic orientation error of approximately 5–6 degrees in these regions. The reported findings have implications for both clinical and research studies based on dMRI FOD measures, and provide an important biological benchmark for improved FOD reconstruction and fiber tracking methods.

## Introduction

Diffusion magnetic resonance imaging (dMRI) [1], [2] is a powerful tool increasingly applied in clinical and research settings for investigating structural properties of biological tissue *in vivo* [3], [4]. The basic concept of dMRI is to quantify the microscopic self diffusion of water along prescribed directions in three-dimensional (3D) space using a series of diffusion sensitive MR images [5]. In biological tissue, the diffusion-driven displacements of water molecules are impeded by intra- and extra-cellular tissue components [6], and therefore their measured displacement distributions provide unique microstructural and architectural information in both normal and pathologic brain tissue [7].

The standard dMRI method is diffusion tensor imaging (DTI) [8], which uses a single 3D Gaussian distribution model for the measured apparent diffusion coefficient (ADC) in each imaging voxel. The shape and orientation of the Gaussian distribution is fully specified by its covariance matrix, or diffusion tensor (DT). In coherent, densely packed, white matter fiber bundles, the direction of fastest diffusion, given by the principal axis (or primary eigenvector) of DT, points along the main axis of the fiber bundle and is commonly used to map the trajectory of white matter fiber tracts in the brain [9]–[11]. However, while the orientation of the DT has been validated in large fiber bundles with coherent fiber orientations in brain [12]–[14] and myocardial tissues [15]–[17], the tensor model cannot be used to resolve multiple fiber bundles within voxels containing more than one principal direction [18]. Such complex fiber architectures frequently occur both in gray and white matter regions containing crossing or branching fiber tracks, and as a consequence of partial volume effects when different neighboring tissue architectures are included in the same voxel. In both cases, the ADC will have multiple diffusion peaks and the DT no longer provides an accurate mathematical description of the apparent diffusion patterns.

This limitation of DTI has prompted numerous efforts to develop dMRI techniques capable of resolving complex fiber architectures within voxels. Diffusion spectrum imaging (DSI) [19] is a popular model-free method that applies the classical formalism of “*q*-space” theory [20], [21] to recover the three-dimensional (3D) diffusion propagator, or displacement spectrum in each voxel. The orientation structure of the diffusion propagator can be revealed by summing the propagator in the radial direction, yielding a measure that quantifies the overall likelihood of water diffusion in a given direction in 3D space. This derived function, called the water diffusion orientation distribution function (ODF) can be used as a surrogate measure of complex fiber orientations within voxels [19], [22]. A related model-free method called *q*-ball imaging (QBI) [23] provides an alternative approach for recovering the diffusion ODF in each voxel using less time intensive and reduced encoding (spherical) diffusion acquisition protocols.

While DSI and QBI are established model-free techniques for recovering important aspects of the water diffusion function in tissue (through measurement of the diffusion propagator or ODF), they do not provide a direct quantitative description of the underlying distribution of fibers or the intrinsic diffusion properties of these fibers [24]. This additional level of inference requires a biophysical model for the diffusion properties of the tissue fibers. One popular model-based method is to model the diffusion function of the neuronal fibers with a single diffusion tensor, where the parallel and perpendicular diffusivity of the tensor is fixed for all fibers within the voxel. Under this model, the intrinsic neuronal fiber orientation distribution (FOD) can be estimated via spherical deconvolution of the diffusion signal with a tensor response function [24]–[29] or using more sophisticated Bayesian methods [30], [31]. Similar to the water diffusion ODF, the peaks of the FOD can reveal the orientation structure of complex fiber architectures within voxels and is gaining popularity for use in fiber tracking applications [25], [32]. Yet another model-based approach is to impart a composite model for the restricted and hindered water inside and outside the myelinated axons to obtain estimates of white matter fiber orientations [33] and diameter distributions [34], [35] within voxels.

To date, validation of the orientation structure of both the water diffusion ODF and underlying FOD have primarily involved numerical simulations in conjunction with qualitative comparison with known anatomical features [25]–[29], [36]–[38], and comparisons with *ex vivo* biological [19], [39]–[41] and non-biological [39], [42]–[44] diffusion phantoms. Thus, there is a lack of direct quantitative validation against realistic biological fiber architectures, and this poses an important limitation to the further development of improved FOD methods and studies which seek to apply these methods for research and clinical purposes.

To amend this, we here quantitatively compare FOD measures derived from *ex vivo* dMRI data against histological measures of rat brain myeloarchitecture. We conclude that FOD measures derived from tomographic dMRI data provide an accurate characterization of underlying myelinated fiber orientation distributions, even in regions with complex fibers architectures where the application of DTI is limited.

## Results

High b-value *q*-space imaging (QSI) data were acquired from fixed adult rat brains that had been immersed in a contrast enhancing gadolinium solution (Magnevist® [45]). Following tomographic imaging, coronal histological sections of the tissue were obtained and stained for myelin and high resolution digital images acquired. Tomographic and histological images from corresponding regions-of-interest (ROIs) were co-registered using affine transformations and quantitatively compared.

### ROI specification

**Figure 1:**
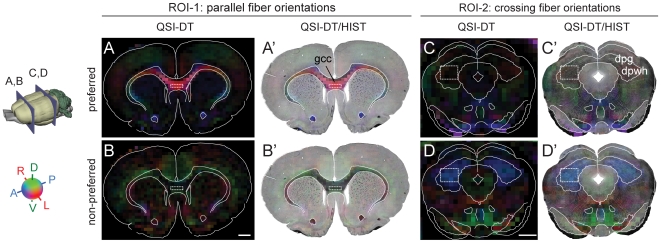
Selection of regions-of-interest. Anatomical ROIs containing coherent or crossing fiber orientations without anterior-to-posteriorly orientated fibers were identified using red-green-blue (RGB) maps of the preferred (**A,A**′,**C**,**C**′) and non-preferred (**B,B**′,**D,D**′) diffusion direction, indicated by the first and third eigenvector of the DT, respectively. Voxel colors indicate the direction of the respective eigenvectors (cf. color code insert, bottom left), while the voxel brightness is given by the degree of linear and planar diffusion anisotropy for the preferred and non-preferred maps, respectively. (**A**,**B**) Coronal RGB maps through the genu of the corpus callosum, and (**C**,**D**) coronal RGB maps through the brain stem at level of the superior colliculus (position indicated on the 3D rat brain insert). (**A**′–**D**′) Overlay of RGB maps and corresponding myelin stained section images. The bright red color in (**A**,**A**′) indicates a high degree of linear anisotropy with left-right orientation, consistent with coherent mediolaterally oriented commissural fibers in this region (see, also Fig. 2C). In the corresponding non-preferred map (**B**,**B**′), the same voxels are dark, indicating a lack of planar diffusion anisotropy. The bright blue color of the non-preferred map in (**D**,**D**′) indicates a high degree of planar anisotropy (typical of crossing fibers, see, also Fig. 2D) within the coronal plane. In the corresponding preferred map (**C**,**C**′) these voxels are dark, consistent with a lack of uniformly oriented fibers. For our quantitative analysis, we selected four voxels in a 1×4 grid in the bright red region in the corpus callosum (ROI-1; dotted white frame in **A**,**A**′,**B**,**B**′) and twelve voxels in a 3×4 grid in the bright blue region in the tectum (ROI-2; dotted white frame in **C**,**C**′, **D**,**D**′). dpg, deep gray layer of the superior colliculus; dpwh, deep white layer of the superior colliculus; gcc, genu of the corpus callosum. Scale bars, 1 mm.

**Figure 2:**
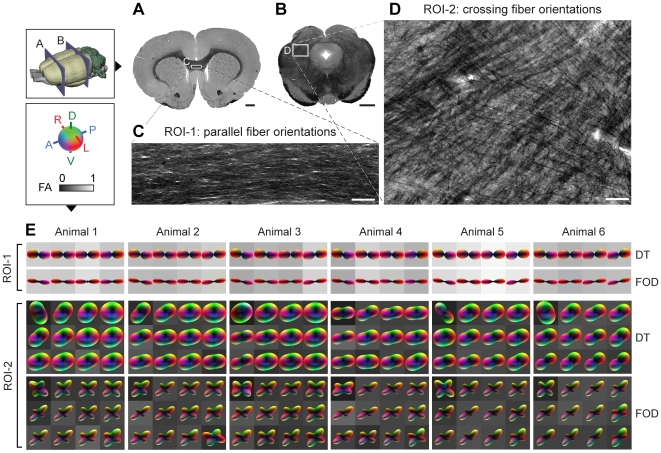
Three-dimensional DT and FOD reconstructions in brain regions with parallel and crossing fiber orientations. (**A–D**) Detailed visualization of the parallel (**A**,**C**) and crossing (**B**,**D**) myeloarchitecture in ROI-1 and -2. (**E**) Comparison of 3D DT and FOD reconstructions from ROI-1 and -2 across all six animal specimens. Reconstructions are overlaid on a gray scale map of the fractional anisotropy (FA) index [55] derived from the DT eigenvalues and quantifying the overall degree of diffusion anisotropy on a 0–1 scale (1 being highly anisotropic and 0 being isotropic). The high FA values and elongated DT and FOD profiles in ROI-1 are characteristic of coherent (parallel) fiber orientations, while the low FA values and disk-like DT profiles in ROI-2 are characteristic of crossing fibers, which is further evidenced by the FOD reconstructions and myeloarchitecture. All DT and FOD reconstructions are shown min-max normalized for reasons discussed in the main text. Scale bars, 1 mm (**A,C**) and 100 µm (**C,D**).

Our first objective was to identify suitable ROIs in which 3D FODs can be compared to inherently 2D, coronally sectioned histological data. We focused our analysis on two ROIs with little or no through-plane (anterioposteriorly oriented) fiber orientations containing 1) coherently oriented fibers, or 2) more complex crossing fiber architectures where the DT model is known to be insufficient. The ROIs were selected on the basis of color-coded DT maps, 3D reconstructions of the DT and FOD, and visual inspection of myelin stained histological sections (Figs. 1, 2). The first region selected (ROI-1) was a 1×4 voxel grid within an area of the anterior part (genu) of the corpus callosum containing high densities of within-plane, mediolaterally oriented commissural fibers [46]–[48]. These orientations are apparent from red colored voxels in the color-coded DT maps (Figs. 1A,B). Because the DT model has previously been validated in regions with uniform fiber orientations [12]–[15], this region was used primarily as a benchmark to confirm that both the DT and FOD provided accurate assays of myeloarchitecture in this region. The second region selected (ROI-2) was a 3×4 voxel grid within an area of the deep gray and white layers of the superior colliculus containing transversely oriented bundles of intratectal and tectal afferent and efferent projection fibers [49], [50] (Figs. 1C,D; 2D). Here the color-coded DT maps showed planar “disk-like” diffusion profiles indicative of crossing fibers oriented within in the coronal plane (Fig. 1D. blue color). In both ROIs assessment of the (coronal) histological sections confirmed presence of predominantly in-plane oriented myelinated fibers (Fig. 2C,D), suitable for two-dimensional histological analysis of fiber orientations.

### Qualitative comparison of DT, FOD and myeloarchitecture

Our second objective was to assess the resemblance of DT and FOD measurements with histological visualizations of myeloarchitecture in the selected ROIs. In ROI-1, 3D DT and FOD reconstructions across all six animals showed the expected “cigar-like” profiles with left-right orientation (Fig. 2E). In ROI-2, 3D DT reconstructions in all six animals confirmed the “disk-like” shape of the DT, and 3D FOD reconstructions indicated the expected crossing fiber patterns (Fig. 2E). The orientation of the FOD reconstructions were highly consistent across animals in both ROIs and with the morphology evidenced in the myelin stained sections (Fig. 2C,D). Taken together, these results demonstrate a high qualitative resemblance between 3D FOD measures and histological observations of crossing fibers which is highly consistent across several animals. To provide a more rigorous quantitative histological validation of this relationship, we continued to perform a detailed voxel-wise quantitative comparison between these measures in ROI-1 and -2 in one of the animal specimens (animal 1).

### Quantitative histological validation of FOD estimates

Our third objective was to quantitatively evaluate the correspondence between high b-value QSI-derived FOD measures (QSI-FODs) and myeloarchitecture in both ROI-1 and 2. As a benchmark for the comparisons we also included DT fiber orientation estimates (DT-FOD) in both regions (see also “Computation of DT-FODs” in the “Materials and Methods” section).

**Figure 3:**
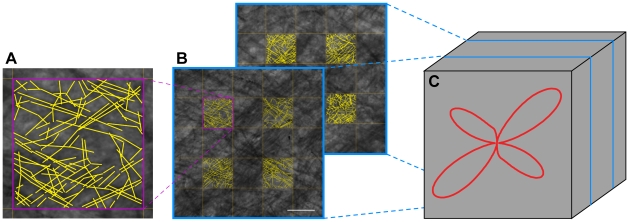
Computation of a single HIST-FOD from multiple myelin stained section images. (**A**) Myelin fiber orientations were manually traced as vector lines within 53×53 µm sample grids positioned above high-resolution images of myelin stained sections. (**B**) For each histological section, 4 sample grids were systematically positioned within a voxel domain. (**C**) For each QSI voxel, myelin fiber orientations were estimated from vector data collected from 8 sample grids across two histological sections spaced at 100 µm. This procedure was repeated for all voxels in both ROI-1 and 2. Scale bar, 50 µm.

To validate the QSI-FODs, we used a voxel-wise stereological sampling approach to manually record several hundred myelin stained fiber orientations from multiple registered myelin stained histological images (Fig. 3A,B). The angular histogram of all fiber samples (or fiber counts in a given angular bin) within each voxel constitutes the empirical histology-derived FOD (HIST-FOD; Fig. 3C) used as the validation standard to compare against the DT- and QSI-FOD estimates. Prior to comparison, all fiber models were min-max normalized to remove the isotropic component and normalize the maximum amplitude of the respective distributions. The isotropic component was removed because in conventional QSI-FOD measures there is no way to separate the isotropic fiber orientation from the isotropic ADC, and thus if not removed, the QSI-FODs would have a (biased) larger isotropic component compared with the HIST-FODs.

**Figure 4:**
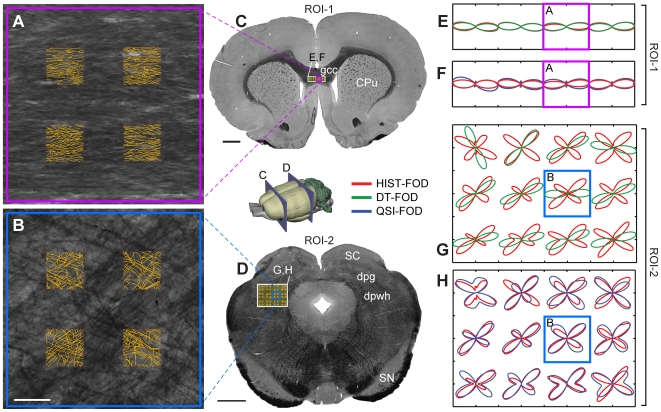
Comparison of FOD estimates against myeloarchitecture. (**A**,**B**) High resolution images of myeloarchitecture corresponding to one QSI voxel with overlay of 4 sample grids of manually-recorded fiber directions (yellow lines) used in part to compute HIST-FODs (8 total sample grids were used, cf. Fig. 2). Purple and blue frames indicate corresponding voxel locations across panels. (**C**,**D**) Coronal myelin sections through the genu of the corpus callosum (**C**) and superior colliculus (**D**) (levels indicated on 3D rat brain insert) showing the position of the 1×4 voxel ROI-1 and 3×4 voxel ROI-2 (white frames, cf. Fig. 1). (**E**,**F** and **G**,**H**) Comparison of QSI-FODs and DT-FODs against corresponding HIST-FODs (**E**,**F**: ROI-1and **G**,**H**: ROI-2). gcc, genu of the corpus callosum; dpg, deep gray layer of the superior colliculus; dpwh, deep white layer of the superior colliculus. Scale bar, 50 µm **(B)** and 1mm (**C**,**D**).

We found that in ROI-1, which contained fibers with relatively homogeneous fiber orientations, both the QSI-FOD and DT-FODs correlated well with the HIST-FODs (average Pearson correlation coefficients r = 0.98; SD = 0.02 for QSI-FODs, and 0.99; SD = 0.01 for DT-FODs, *n* = 4 voxels; Fig. 4E,F). By contrast, in ROI-2, which contained crossing fibers, the DT-FODs correlated poorly with HIST-FODs (average Pearson correlation coefficients r = 0.16; SD = 0.38; *n* = 12 voxels), while QSI-FODs correlated substantially better (average Pearson correlation coefficients r = 0.86; SD = 0.07; Fig. 4GH). A non-parametric permutation-based 2-sample t-test revealed that the difference in correlation between the DT-FODs and QSI-FODs were not significant in ROI-1 (p≫0.05), but highly significant in ROI-2 (p<0.0001). These results quantitatively confirm that QSI-FODs provide accurate assays of the underlying myeloarchitecture in regions of both uniform and complex crossing fiber orientations.

**Figure 5:**
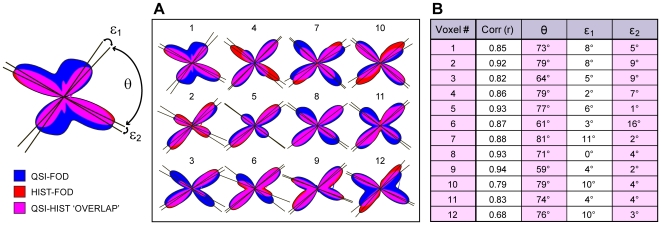
Quantitative comparison of fiber orientation distributions in ROI-2. (**A**) Superimposed HIST-FODs (red) and QSI-FODs (blue) are shown for each of the 12 voxels in ROI-2, together with peak orientations (black lines) for the respective fiber orientation distributions. Purple color indicates FOD overlap. (**B**) For quantitative analysis, the acute intersection angle of the HIST-FODs (θ), and the angular error of both FOD peaks (ε_1_ and ε_2_) are given for each of the 12 voxels together with the Pearson correlation coefficients (r) between the HIST- and QSI-FODs. The average Pearson correlation was r = 0.86; SD = 0.07, average intersection angle was θ = 73°; SD = 8°, average angular error (ε_1_ and ε_2_ combined) was 5.7°; SD = 3.8°.

We also quantitatively evaluated the orientation error of the QSI-FOD peaks in ROI-2 (Fig 5). We found that the peak orientations of the QSI-FODs closely matched the peaks of the HIST-FODs, with an average angular error (across all 12 voxels) of 5.7°; SD 3.8° (Fig 5). The average (acute) intersection angle of the HIST-FOD peaks was θ = 73°; SD = 8° (Fig 5). It should be noted that these results were obtained using a radial-basis function parameterization of the QSI-FODs with a width of σ = 10 (see “Materials and Methods”), which maximized the correlation with the HIST-FODs in ROI-2. However, further testing revealed that the angular error and correlation were relatively robust to variations in σ. When testing a range of σ between 1 and 20, the average angular error and correlation coefficient only varied between 5.4°–6.2° and 0.83 and 0.86, respectively (data not shown).

## Discussion

While methods for estimating neuronal fiber orientation distributions (FODs) in dMRI are becoming increasingly popular, the correspondence between FOD measures and realistic biological fiber architectures has been unclear. Using detailed manual recordings of individual myelin stained fiber orientations in *ex vivo* rat brain tissue we have shown that tomographic dMRI FOD estimates provide accurate assays of the underlying myeloarchitecture, even in regions with complex multi-directional crossing fiber architectures.

In this study, FODs were quantitatively validated using a voxel-wise approach against empirical FOD estimates derived from registered myelin stained images. As evident in Figure 4F,E, QSI-FODs provided an accurate characterization of the underlying myelinated fiber orientation distribution in regions of both uniform (r>0.9) and crossing fiber (r>0.8) architectures. A subsequent evaluation of the angular error of the QSI-FOD peaks in ROI-2 demonstrated an average angular error of approximately 5–6°, with an average (acute) fiber crossing angle of approximately 73° (Fig. 5). Because it seems reasonable to assume that some of this error is likely due to image registration and stereological errors (e.g., through-plane fiber contributions and sampling limitations), the actual intrinsic angular error of the QSI-FOD peaks is probably even less than 5–6°.

It should be noted that the FODs in this paper were computed from QSI data with high b-values and a large number of diffusion measurements (b-max = 30452 sec/mm^2^, 515 diffusion measurements, see “Materials and Methods”). Thus, it remains to be determined to what extent similarly accurate assays of myeloarchitecture can be derived using more conservative spherical acquisition protocols as often employed on clinical 1.5T and 3.0T scanners for *in vivo* applications. However, numerous simulation studies [44], [51] have demonstrated that FODs (with the ability to resolve crossing fibers down to about 45°) can be derived from spherical acquisition protocols using moderate b-values (b∼2000–4000 s/mm^2^) and reasonable scan times (SNR∼30, scan time<10 min). These simulation studies suggest that accurate FODs can be achieved in regions with complex architectures with use of reduced encoding spherical acquisition protocols. Hence, the validation benchmarks established in this paper will likely also have a high degree of translational value for the clinical situation.

The histological FODs were based on manually traced myelin fibers sampled from high-resolution digital images of 50 µm thick sections using a systematic random approach. Sampling occurred both within (using sampling bins) and across planes (one focal plane in two sections spaced at 200 µm) to ensure that a representative fraction of the myeloarchitecture was recorded per voxel volume. It should also be noted that water diffusion is influenced by the complete tissue microarchitecture and not only myelinated fibers (for review, see Ref [6]), which will bias the dMRI measurements relative to the histological measurements. However, the high correlations measured suggest that the contribution of other tissue elements is relatively small in the regions investigated.

The QSI-FODs showed a remarkable consistency across animal specimens (Fig. 2E). We thus chose to restrict the quantitative validation to extensive, in depth anatomical analyses of fiber architectures in one specimen. In these analyses, FOD estimates within each voxel were derived from several hundred manually traced histological measurements across multiple coronal sections and several hundred QSI diffusion measurements. Thus, although the total number of voxels compared was relatively small (4 for ROI-1 and 12 for ROI-2), the statistical correlations were based on measurements derived from extremely high dimensional datasets. It should further be noted that the statistical correlations were only used to provide a quantitative metric of similarity at each voxel, and not as a statistical test of consistency or generalizability across animal specimens.

The voxel-wise histological validations were conducted in paraformaldehyde fixed tissue that had been immersed in contrast enhancing Magnevist® liquid (see “Materials and Methods”). Because this treatment is known to reduce the ADC [45], some care should be exercised when extrapolating these results to the *in vivo* case. However, it has been shown that fixation has relatively small effects on the overall amount of diffusion anisotropy, as the ADC is reduced equally in all directions [45]. Therefore, the fixation process itself is not likely to have influenced the general orientation structure of the QSI-FOD measurements in this study.

We conclude that fiber orientation distributions derived from high dimensional diffusion MRI data provide accurate assays of the underlying myeloarchitecture, even in regions with complex crossing fiber architectures. These results have important implications for both clinical and research studies investigating structural aspects of biological tissues using estimates of the fiber orientation distribution. Furthermore, this study provides an important biological benchmark for further improvement of fiber orientation reconstruction and tracking methods.

## Materials and Methods

### Ethics statement

Animal procedures were approved by the institutional animal welfare committee at the Massachusetts General Hospital, and were in compliance with National Institutes of Health guidelines for the use and care of laboratory animals.

### Material and data acquisition

Adult male Sprague-Dawley rats were anesthetized (ketamine hydrochloride 50 mg/kg, and sodium pentobarbital 12 mg/kg, i.p.) and euthanized by transcardial perfusion with 4% paraformaldehyde. The isolated brains were immersed for 4 weeks at 4°C in a solution of 1mM Gd-DTPA (Magnevist®, Bayer HealthCare Pharmaceuticals, Wayne, NJ, USA) in phosphate buffered saline, and positioned in a sealed plastic tube filled with Fomblin® LC8 liquid (Solvay Solexis, Thorofare, NJ, USA) [45]. High b-value QSI data were acquired using a 2D spin echo planar imaging (EPI) sequence on a 4.7T Bruker scanner equipped with a 3 cm solenoid receiver coil. QSI data were collected using a conventional DSI (Cartesian) acquisition scheme. Pulse-sequence parameters for the QSI acquisition were: TR/TE = 650/49 msec, Δ/δ = 23/12 msec, 515 q-space directions, |G|_max_ = 380 mTm^−1^, b-max = 30452sec/mm^2^, matrix = 64×64×128, voxel size = 265 µm isotropic. Following MR imaging, the brain was coronally sectioned at 50 µm on a freezing microtome, at an angle closely matching the tomographic images. The right side of the brain was marked with a shallow razor-blade cut in the tissue to ensure correct orientation of the sections. One in four sections was stained for myelin using a standard procedure modified from Woelcke [52], yielding an effective through-plane spacing of 200 µm. High-resolution mosaic images were obtained through UPlanApo 20/0.70 and 40/0.85 dry objectives using a motorized Olympus BX52 microscope running the Neurolucida 7.0 software (Virtual Slice module, MBF Bioscience, Inc, Williston, VT, USA).

### Registration

As any voxel-wise quantitative comparison requires accurate spatial registration of image data, several measures were taken to minimize the potential error of misalignment. First, care was taken during histological processing to ensure that the coronal sectioning angle matched the tomographic slice orientation. Trigonometric measurements of multiple corresponding anatomical landmarks confirmed that the angle of the histological section plane and tomographical slice orientation only differed by about 2 degrees (rotation around the mediolateral axis), thus allowing direct registration without resampling of the QSI slice orientation. Second, the selected ROIs (procedure described below) were confirmed to have 1) minimal nonlinear distortion in both the histological data (due to histological processing) and QSI data, and 2) consistent myeloarchitecture across multiple coronal (through-plane) sections. Third, a careful and detailed manual registration protocol was performed for both ROIs. Corresponding anatomical landmarks (brain surface, genu and splenium of the corpus callosum, anterior commissure, the ventricular system, the oculomotor nerve, and several mesencephalic and brain stem nuclei) were identified on basis of general gray and white contrast and used to assign relative anterioposterior position coordinates across the whole brain for both image modalities. Then, for each ROI (one in the forebrain at the level of the genu of the corpus callosum, and the other in the brain stem at level of the superior colliculus, see Fig. 1), histological images were manually registered to corresponding QSI images. Groups of corresponding tomographical and histological images from a volume approximating a ∼1 mm thick coronal brain section (corresponding to 4 QSI and 20 histological slices) through both ROIs were assembled as separate layers using the program Adobe Illustrator CS3 (Adobe Systems Inc. San Jose, CA, USA). Each layer was scaled appropriately depending upon the native voxel size. Finally, using the QSI images as a reference, the histological images were individually adjusted using affine transformations to match multiple local gray and white matter landmarks. This final alignment procedure was iterated until optimal spatial matching was achieved.

### ROI selection

ROIs were selected on the basis of 1) color-coded DT maps, 2) 3D DT and FOD reconstructions, and 3) visual inspection of myeloarchitecture. The first ROI (ROI-1) was selected to contain relatively uniform white matter fiber orientations (Figs. 1A,B; 2A). To help find this location, a red-green-blue (RGB) map was created using the primary eigenvector 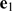 of the DT (“preferred” direction), where each vector element defined the red, green, and blue components of each voxel, respectively. The brightness of this preferred RGB map was determined using a measure of the degree of linear anisotropy, defined as *C_l_* = (λ_1_−λ_2_)/λ_1_ [53], where 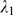, 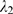, and 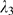 are the first, second, and third eigenvalues of DT, respectively. Thus, a bright red color in the preferred RGB map indicates a high degree of linear anisotropy with left-right orientation (Fig. 1A,A′). The second ROI (ROI-2) was selected to contain complex crossing fiber orientations (Figs. 1C,D; 2B). To help find this location an RGB map of the third eigenvector 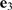 (“non-preferred” direction) was created and the brightness of this non-preferred RGB map was defined using a measure of the degree of planar anisotropy, defined as *C_p_* = (λ_2_−λ_3_)/λ_1_ [53]. Voxels with a bright blue color would thus indicate a “disk-like” DT (indicative of crossing fibers) within the image plane (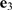 points through-plane, Fig. 1D,D′). 3D DT and FOD reconstructions provided additional confirmation of apparent crossing fiber architectures that were highly consistent across animals. Finally, the myeloarchitecture in ROI-1 and ROI-2 were microscopically inspected in the original histological sections to confirm the neuroarchitectural patterns suggested by the tomographic data.

### Computation of HIST-FODs

The HIST-FODs in ROI-1 and 2 were derived using a systematic random stereological approach adapted from [12]. High-resolution histological images from two consecutive myelin stained sections (spaced at 200 µm) were assembled in separate layers in the Adobe Illustrator file used for image registration (see registration section above). A grid derived from the voxel matrix of the spatially corresponding QSI slice was superimposed onto the histological images. Each voxel domain was further subdivided using a 5×5 rectangular sampling grid. In four systematically positioned sample grids (Fig. 2A,B), individual myelin fiber trajectories were traced as vector lines using Adobe Illustrator. The vector coordinates were exported to Matlab (The Mathworks, Inc. Natick, MA) and HIST-FODs were computed for each QSI voxel by calculating the angular histogram of the line plots (fiber vectors) with a bin size of 1° (360 points) from the four sub regions. To further increase the robustness of the HIST-FODs, corresponding angular histograms from two neighboring myelin sections spaced 200 µm apart were averaged together and the averaged histogram was smoothed with a Gaussian kernel with a FWHM of 8°. In this way, our histological sampling included a through-plane distance approximately corresponding to the depth of a single 265 µm QSI voxel. To quantify the intrinsic degree of fiber spreading within ROI-1 of the corpus callosum, Gaussian distributions were fit to the final angular histograms of each voxel, yielding an average FWHM of approximately 34°.

### Computation of DT-FODs

Since the diffusion tensor (DT) is a model for the apparent diffusion coefficient (ADC), a direct comparison with the HIST-FODs would be misleading. Under the DT model, the fiber orientation is given by a single delta function pointed in the direction of the primary eigenvector. To generate DT equivalent FODs (DT-FODs), the resultant delta functions were convolved with a Gaussian smoothing kernel with a FWHM of 34°. This level of smoothing was chosen to match the intrinsic angular dispersion of fibers within the ROI-1 of the corpus callosum.

### Computation of QSI-FODs

To estimate the QSI-FODs, we extend the traditional spherical deconvolution method in Reference [29] to arbitrary (multi-b-value) *q*-space acquisitions. An axially symmetric tensor model [54] was chosen for the single fiber response function with perpendicular (λ_⊥_ = λ_2_ = λ_3_ = 4.0×10^−5^ mm^2^s^−1^) and parallel diffusivities (λ_∥_ = λ_1_ = 3.5×10^−4^ mm^2^s^−1^) estimated directly from the QSI data in ROI-1, similar to the approach used in [29]. Tikhonov regularization was used to improve the conditioning of the inversion. The FOD solution was parameterized using radial-basis functions, as in Ref [26]. The radial-basis functions themselves are parameterized by the desired FOD reconstruction points and the radial-basis function width parameter σ. For the 3D QSI-FOD reconstructions in Figure 2, a 3^rd^ order icosahedral tessellation of the sphere was chosen for the FOD reconstruction points (642 vertices) and σ was set to 20. For the 2D QSI-FOD reconstructions in Figures 4 and 5, 360 equally spaced points on the unit circle was chosen for the FOD reconstruction points, and σ was set to 10. This value of σ was chosen because it optimized the correlations with the HIST-FODs in ROI-2. However, we tested a range of different values for σ (from 1 to 20) and found that the average correlations and angular error for the FOD peaks were largely robust to variations in this parameter.

### Statistical analyses

Data plotting and correlation analyses were performed using the standard Matlab statistical toolkit. Correlations were given as Pearson correlation coefficients and where appropriate standard deviations are provided.

### Illustrations

Graphical charts were generated using Matlab. Figures were assembled using Adobe Photoshop (CS3) and Adobe Illustrator (CS3).
